# Research Priorities for Preconception Health in Scotland: A Multidisciplinary Call to Action

**DOI:** 10.1111/hex.70915

**Published:** 2026-10-29

**Authors:** Sinead Currie, Emma J. Brough, Laura Ryan, Kathleen Renwick, Beth Rose, Annie Ganguly

**Affiliations:** ^1^ Psychology, Faculty of Natural Sciences University of Stirling Stirling Scotland UK; ^2^ Department of Built Environment and Life Sciences, Faculty of Social and Applied Sciences Abertay University Dundee Scotland UK; ^3^ ASH Scotland, Engagement Team Edinburgh Scotland UK; ^4^ NHS National Research Scotland Aberdeen Royal Infirmary, Foresterhill Aberdeen Scotland UK

**Keywords:** preconception health, public and patient involvement (PPIE), research priority setting

## Abstract

**Introduction:**

Health and behaviours prior to conception influence reproductive outcomes, lifelong health, and health inequalities across generations. While international attention to preconception health has increased, context‐specific evidence to inform effective practice and policy in Scotland remains limited. This gap is concerning given declining birth rates, widening inequalities, and increasing prevalence of adverse maternal and infant outcomes.

**Methods:**

A research prioritisation project was undertaken to identify key areas for preconception health research in Scotland. A narrative review of published literature reviews and relevant Scottish policy and public health guidance was conducted to identify evidence gaps and preliminary research themes. These informed a national online survey that captured views from members of the public and stakeholders with an interest in preconception health. Survey findings were subsequently refined through an in‐person, multi‐stakeholder workshop involving members of the public, researchers, healthcare professionals, third sector representatives, and policy stakeholders.

**Results:**

Four overarching research priority themes were identified: (1) adopting a life‐course approach to preconception health; (2) reducing neurodevelopmental risks and improving awareness of preconception behaviours; (3) addressing health inequalities and ensuring equitable access to care; and (4) enhancing the involvement of men, partners, and shared responsibility for preconception health. Cross‐cutting principles emphasised the need for inclusive and consistent messaging, improved healthcare professional training, integration of preconception care within existing services, stakeholder involvement, and robust data collection.

**Conclusion:**

This co‐designed research prioritisation project identifies clear and actionable priorities for advancing preconception health research in Scotland. Addressing these priorities will support evidence‐based policy and practice, improve reproductive and long‐term health outcomes, and contribute to reducing health inequalities for current and future generations.

**Public Contribution:**

Members of the public and individuals with lived experience of preconception health were actively involved throughout this research prioritisation project. Public contributors participated in a national survey to identify and rank priorities for preconception health research in Scotland and attended a multi‐stakeholder workshop to refine and finalise research themes and questions. The workshop included members of the public alongside researchers, healthcare professionals, third sector representatives, and policy stakeholders, ensuring that priorities reflected lived experience as well as professional perspectives. Public input directly informed the identification of research priorities, the interpretation of findings, and the development of future research directions (See the GRIPP2 short form checklist in the supplementary files).

## 1 Introduction

The health and health behaviours of individuals before conception (preconception) impact lifelong health and health risks. For example, maternal obesity before conception is associated with 264% higher odds of childhood obesity [1], with a comparable association for paternal obesity [2]. Maternal smoking before pregnancy triples the risk of congenital heart defects [2]. Whereas pre‐pregnancy folic acid supplementation reduces the risk of congenital abnormalities by 43% [2]. Therefore, supporting women and men to optimise health behaviours before pregnancy presents an opportunity to improve subsequent health outcomes for parents and children for current and future generations.

Preconception care comprises biomedical, behavioural, and social health interventions delivered before conception to improve maternal and child health outcomes [3]. Understanding preconception health and care requires perspectives from a range of disciplines, including biology, medicine, psychology, behavioural science, epidemiology, sociology, public health, environmental science, nursing, health visiting and maternity care. Therefore, research aimed at understanding the mechanisms that reduce risks and improve outcomes in the preconception period is inherently multidisciplinary.

### 1.1 Preconception Health and Care Research

A seminal series published in the Lancet in 2018 [4, 5, 6] and subsequent publications in 2026 [7, 8] have helped to raise awareness of preconception health and its potential impact on societies. However, there remains to be a significant gap in preconception health research, specifically in the generation of context‐specific, high‐quality research evidence which can be applied into practice and robustly evaluated [7, 8].

Indeed, while a range of high‐quality research exists from Europe and further afield, research involving the Scottish population and reflecting the Scottish health service is lacking. The most up‐to‐date birth rate in Scotland, for the year 2024, was 45,763 births, published by the National Records of Scotland [9]. This continues the trend of decreasing numbers of live births in Scotland since the last peak in 2008 [9]. With an ageing population and the lowest fertility rate in the United Kingdom [10], it is vital to ensure that each baby born in Scotland is as healthy as possible.

### 1.2 Preconception Health in Scotland

Recent Scottish maternity data further underscore the importance of improving preconception health and addressing inequalities [11]. In 2024/25, almost a quarter (24.6%) of maternities occurred among women living in the most deprived areas, with deprivation consistently associated with poorer outcomes across the maternity pathway. Over one‐third (33.5%) of live singleton births followed induction of labour, with substantial variation across NHS boards and a higher likelihood among women from more deprived areas. Maternal obesity (28.5%) and diabetes (9.8%) remain prevalent, disproportionately affecting women in deprived communities. Caesarean section rates have continued to rise, reaching 43.5% of live singleton births, while preterm birth rates increased to 6.8%, again showing a clear social gradient. Together, these patterns highlight persistent and widening inequalities in maternal and infant health outcomes in Scotland.

In 2025, Public Health Scotland (PHS) reported that 1 in 58 newborn babies born had a congenital condition, many of which are preventable through adequate maternal nutrition during the preconception and early pregnancy periods [12]. It is inappropriate to place responsibility solely on mothers, particularly in the context of low awareness of preconception risk factors [13]. Poor public health messaging, inadequate timing of distribution of pregnancy vitamins, and the UK government's failure to implement folic acid fortification to a level which could meaningfully reduce risks have a role to play. A situation analysis performed for one health board in Scotland emphasised the need for improved preconception care in Scotland both before and between pregnancies to enhance outcomes for women and infants [14]. Given Scotland's devolved powers for healthcare and education, research in this area is urgently needed.

Noting these stark statistics and despite the limited preconception health research from Scotland, the Scottish Government have acknowledged the fact that preconception health is important and have included it within many important health policies and plans over the past 2 years. For example, Scotland's Population Health Framework 2025–2035, Women's Health Plan: Phase Two (2026–2029), the Delivery Framework for Miscarriage Care in Scotland, and the Programme for Government 2025–2026 [15, 16, 17]. These developments in policy and guidelines are extremely important for setting the agenda to address and improve preconception health however, high‐quality, context‐specific research and funding is required in order to fully support meaningful change and to action the Scottish policies and guidelines.

### 1.3 Stakeholder Involvement

Involvement of key stakeholders in policy and research development is essential to maximise success and appropriate tailoring. In 2025, Healthcare improvement Scotland conducted and published a citizens panel report which included preconception health for the first time. This provided a representative sample of public views on preconception health and care [18]. Findings indicated that most respondents would be comfortable talking about preconception health with a healthcare professional and that partners should be equally involved. Furthermore, when asked what matters most to them, 42% mentioned being in good health while others mentioned access to information and advice (23%), and ensuring the baby's health and safety (15%). This indicates a want and need from the Scottish population for effective and targeted preconception health care and support.

Understandably, funding for health research is limited hence difficult choices are required to allocate funds [19]. This is why research priority setting is essential, to ensure that any available funds are utilised for the most important research and has the biggest impact. Hence, a research prioritisation project is required to identify the key areas of need in order to produce high‐quality, impactful, co‐designed preconception health research from Scotland. Such research will provide the basis for evidence‐based practice, support policy development, identify appropriate interventions, improve healthcare practice and subsequent health outcomes of the population.

### 1.4 Project Aims

This research prioritisation project aimed to co‐design research priorities for preconception health research in Scotland with key stakeholders including representatives of the Scottish preconception population, academics, clinicians, third sector organisations (such as charities, voluntary and community organisations) and policy makers.

## 2 Methods

This project followed principles of research prioritisation as outlined by The James Lind Alliance [20] and Lund et al. [21]. This included (1) narrative review and situational analysis with initial refinement of key areas identified, (2) national survey to collate views and priorities from stakeholders, (3) stakeholder workshop to refine and develop priorities.

### 2.1 Step One: Narrative Review and Situational Analysis

Step One aimed to identify gaps and needs in preconception health research relevant to Scotland (Figure 1). A narrative review of existing literature and relevant Scottish policy and guidance was undertaken.

**Figure 1:**
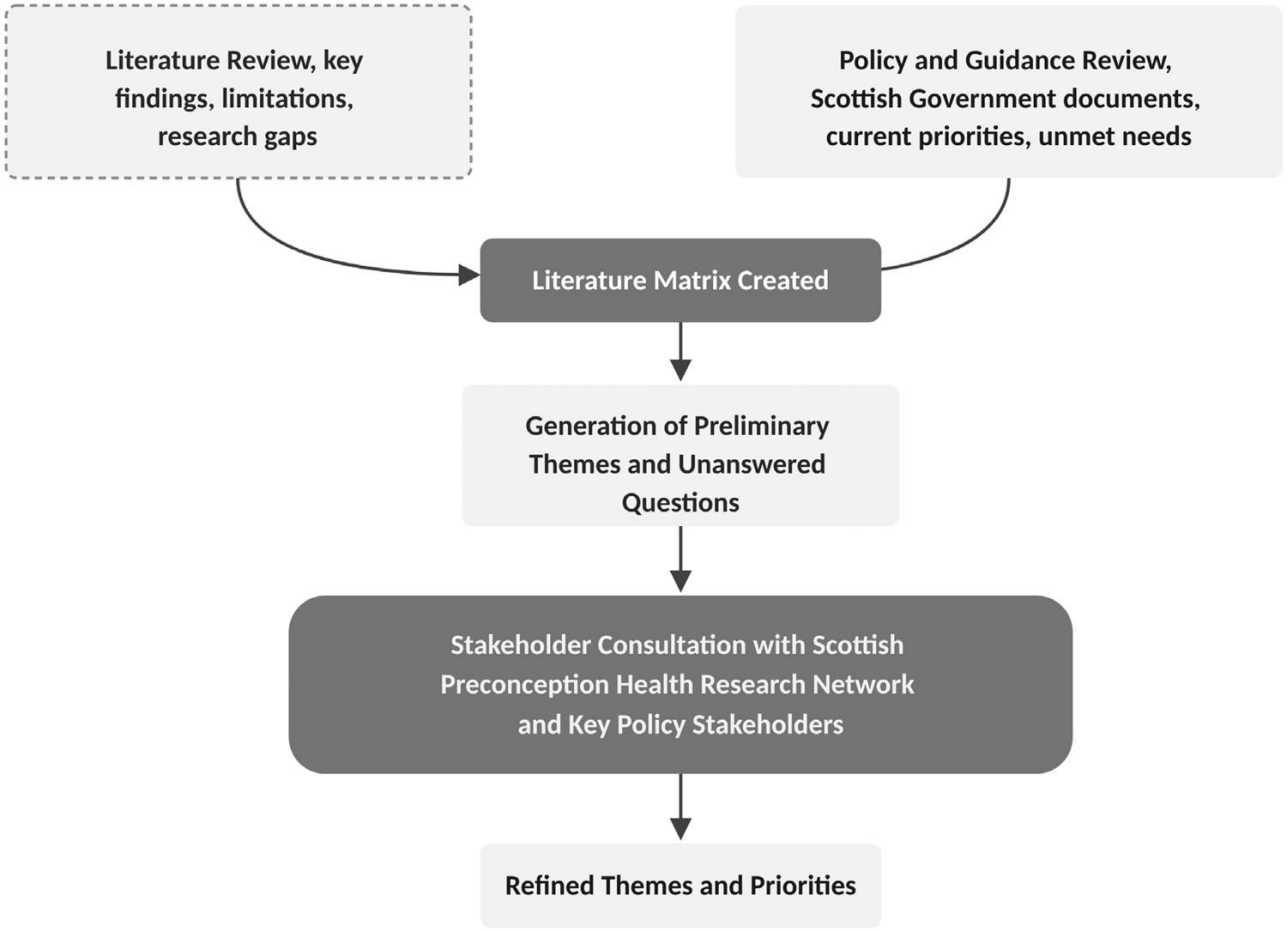
Flow chart of step one (narrative review and situational analysis).

Using CINHAL, a search of peer‐reviewed, published reviews of literature was conducted by the team (S.C., E.B., K.R.) in February 2025. Any review which addressed preconception health and included at least one paper from the United Kingdom was included. All reviews were read, summarised and key data extracted (including review findings, implications, limitations and explicit suggestions for future research). The research team (S.C., E.B., K.R.) then collated the key areas of future research.

In March 2025, official websites, Scottish Government policy documents, public health body reports and grey literature were searched to identify key areas of need for preconception‐related health in Scotland. Only current versions were included to generate an up‐to‐date picture of preconception health research needs for Scotland. Relevant documents were collated, summarised and key data extracted.

The research team (S.C., E.B., K.R.) then collated the key areas of need for preconception‐related health in Scotland. This list of topics was discussed with members of the Scottish Preconception Health Research Network and key policy stakeholders to refine priorities and inform subsequent survey design.

### 2.2 Step Two: National Survey

An online survey collected data from representatives of the Scottish population about their views on preconception health research need. Survey data were collected online using the Gorilla Experiment Builder platform (Gorilla, https://gorilla.sc/), a validated platform for online behavioural and survey research [22].

The survey was aimed at preconception health stakeholders; members of the general public who were 16 years and older living in Scotland and/or those whose work (paid or unpaid) related to preconception health. Ethical approval was granted from University of Stirling General University Ethics Panel (22682). Data were collected between June and October 2025.

Survey content was informed by data from step one. The survey questions can be found in Appendix One. Familiarity of preconception health as a concept and importance of preconception health were answered using a five‐point Likert scale (from one not familiar/important at all to five extremely familiar/important).

Priority questions were answered using Likert scales asking participants to rate whether the theme or question was important, not important or neither. Open‐ended questions were included to ensure participants could contribute additional research themes, questions and opinions.

At the end of the survey, participants were given the researchers contact details and relevant NHS inform webpages for additional information. Participants could also opt in to a prize draw to win 1 of 10, £20 vouchers.

The survey data were analysed by adding the Likert ratings to create a draft ranking of research priorities. Any additional research themes and/or questions from the qualitative data were matched to existing themes and priorities or organised into additional research themes.

The survey was advertised via existing research and professional networks, through social media and by poster advertisement in relevant non‐NHS health and research locations. A recruitment strategy was developed with network members who actively distributed the survey advert to their networks. A wide range of third sector organisations (such as food banks, sexual health charities, parent support groups across many areas of Scotland) were also contacted via email by a member of the research team and asked to disseminate the study advertisement. This was done in numerous phases in an attempt to maximise participant numbers. It was aimed to recruit as close to 1000 participants as possible with an even balance of PPI representatives and others. Potential participants were presented with the online participant information sheet and consent form before the survey was presented to them online.

### 2.3 Step Three: Workshop

An in‐person workshop was held in March 2026. The event aimed to further define research priorities and explore how these priorities could be targeted with future, collaborative research projects. See Figure 2 for flowchart of the methodology. The event was advertised widely via the same channels as the survey and a range of stakeholders were encouraged to attend. This included members of the public, researchers, healthcare professionals, third sector organisation representatives, policy makers, civil servants and anyone doing work related preconception health who would like to help shape the future of preconception health research in Scotland. Students who attended could apply for their travel costs to be reimbursed and members of the public who attended were offered a Public and Patient Involvement and Engagement (PPIE) payment in line with NIHR guidance [23]. Attendance at the event was free. See Appendix S2 for the workshop agenda. The key themes from steps one and two were used as a basis for discussion and research priority development. An independent facilitator led the workshop. The facilitator was a community development practitioner and values‐based leadership facilitator who had no prior involvement in the project or preconception health research. The facilitator ensured all members of the workshop felt comfortable and able to add their views either by voicing them or writing them down. The facilitator provided participants with a range of questions to further refine and prioritise the research themes. Prompts were provided to develop implications, uncertainties, priority topics and funding importance. This content and discussion were finalised into set of priority research topics and research questions by the Scottish Preconception Health Research Network.

**Figure 2:**
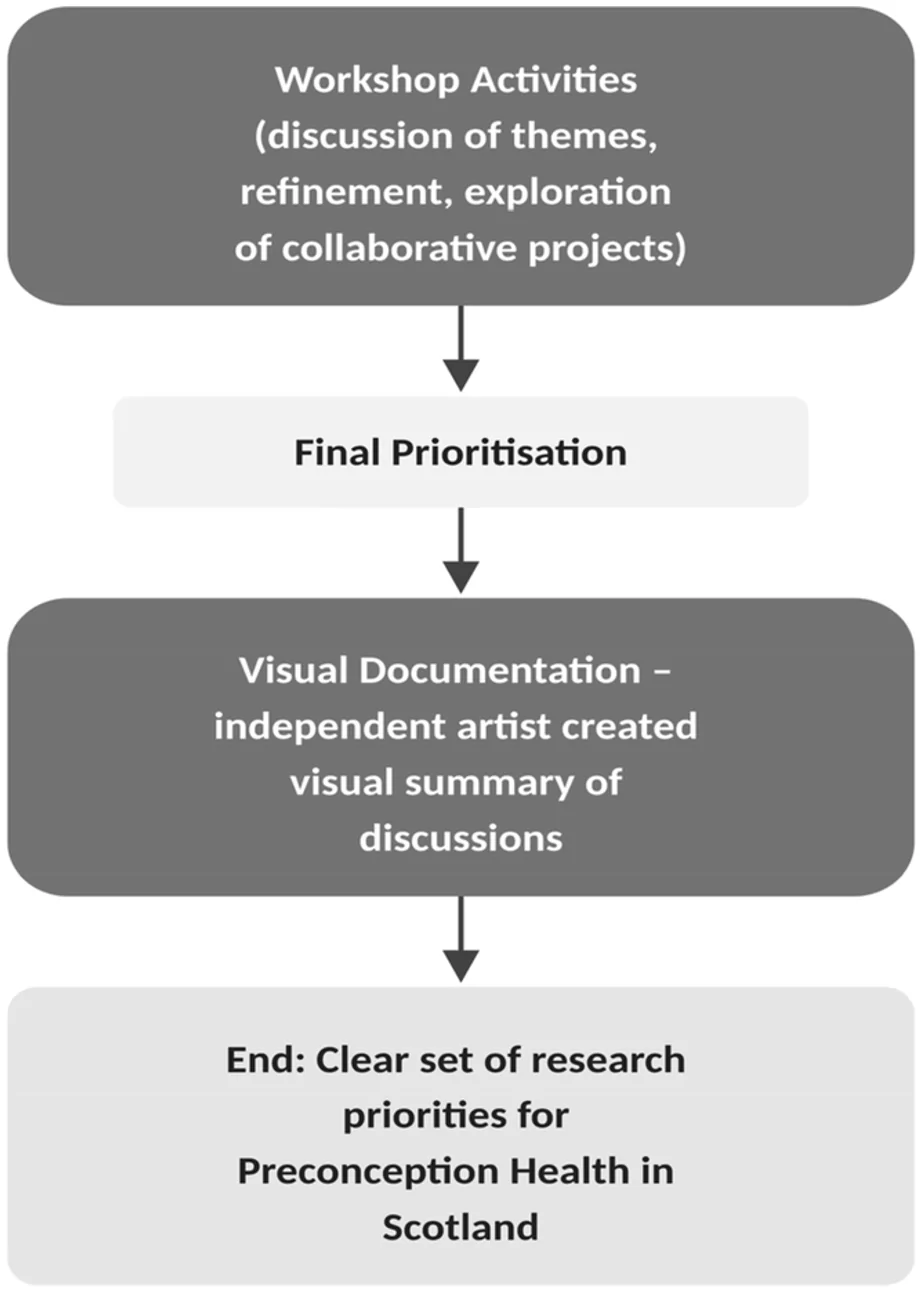
Flowchart of step three (workshop).

An independent artist was also present at the workshop to visually depict the discussion. The artist observed the workshop and produced the piece of work in real time based on observations and content of workshop discussions from stakeholders.

## 3 Findings

### 3.1 Step One: Narrative Review and Situational Analysis

Eleven published peer reviewed reviews and six policies/guidance documents informed this step of the project [24, 25, 26, 27, 28, 29, 30, 31, 32, 33, 34, 35, 36, 37, 38, 39, 40, 41]. All reviews and policy/guidance documents were UK‐based (none were Scotland‐specific) and published between 2015 and 2024, with a focus on policy, guidelines, service provision and interventions for people of reproductive age.

Priority topics were initially derived by the research team from the narrative review and policy analysis (see Figure 1) and subsequently refined through consultation with the Scottish Preconception Health Research Network and key policy stakeholders, resulting in the 36 research questions taken forward to the survey (Step two).

Across the reviews and policy documents, a range of evidence gaps and areas of research need were identified, spanning individual, psychological, social, organisational and cultural factors. These are summarised in Table 1.

**Table 1:** Key topics and areas of research needs from narrative review and situational analysis.

Topic	Area of research need
Sources of information	Online delivery preferred but unclear on detail
	Influence of friends and family needs to be addressed and maximised
Partners, men and women	All parties need to be supported, educated and targeted
	Shared responsibility between men and women is desired but not evident
Psychological determinants	What are motivations for preconception health?
	How can knowledge, intention and behaviour be supported?
	Increasing knowledge base of reproductive health including conception, fertility and ovulation.
	Enhancing empowerment: Beliefs in capabilities to engage in preconception health promoting behaviours.
Societal focus of PC health	Education is needed at all ages regardless of pregnancy intent
	Societal norms and education need to be developed and supported
	There is currently low awareness of epigenetics and personal control
	There is a need for public health involvement to facilitate public awareness of preconception health
	How can preconception health promotion and awareness be capitalised and supported outside of health care?
The intricate and varying cultural beliefs and intricacies related to preconception health	
The most effective and acceptable ways to address and improve pregnancy planning	
A wider behavioural focus is needed including physical activity, environmental issues, stress, sleep	
Systems approach	How can preconception health be interconnected into wider health needs, reproductive health and public health?
	How can individual, organisation and community levels support individual, community and population need?
	Public awareness on a wide scale
Populations	Priority populations to improve preconception health and support include existing chronic illness and metabolic conditions
	How can people with differing pregnancy intentions, differing parity and differing SES be best supported?
Clinical guidelines	Consistency of health guidance is needed
	Quality control is needed i.e. evaluation of guideline application
	Clarity on application is needed e.g. how, where, who's responsibility to deliver preconception health care
	Require adaptability for individuals and services
Preconception care	How can this be best delivered? where, by who, to who, maximising accessibility
	How can preconception care be best integrated into existing services
	What training is needed for health care professionals and those delivering preconception health care?
	Should and could individuals be offered support for pregnancy readiness?
	Capitalising on people's preference for receiving preconception care
	What are the primary care opportunities for delivering preconception health care?
	How can preconception care be promoted to facilitate equal access?
Research methods	More representative samples required
	Longitudinal data required

### 3.2 Step Two: Survey

A total of 104 participants completed the survey. Table 2 shows survey demographics for those who completed each question.

**Table 2:** Participant demographics from survey.

	*N*	%
Age		
16–24	10	9.6
25–34	30	28.8
35–44	27	26.0
45–54	24	23.1
55–64	10	9.6
65+	3	2.9
Total	104	100
Gender		
Female	96	92.3
Male	8	7.7
Total	104	100
Sexual Identity		
Bisexual	11	10.5
Heterosexual	87	83.7
Homosexual	3	2.9
Other	1	1.0
Prefer not to say	2	1.9
Total	104	100
Area (grouped by NHS Scotland boards)		
Aberdeen	12	11.5
Dumfries and Galloway	1	1.0
Tayside	6	5.8
Lothian	17	16.3
Forth Valley	20	19.2
Borders	6	5.8
Greater Glasgow and Clyde	20	19.2
Highland	4	3.9
Ayrshire and Arran	9	8.7
Fife	2	1.9
Lanarkshire	5	4.8
Missing	2	1.9
Total	104	100
Ethnicity		
Arab	1	1.0
Asian (including Scottish or British)	6	5.8
Mixed or multiple ethnic group	3	2.9
White (including Scottish, British and other)	80	76.9
Prefer not to say/missing	14	13.5
Total	104	100
Education		
Degree or higher	84	80.8
Further education	10	9.6
Upper school	7	6.7
Lower school	2	1.9
No qualifications	1	1.0
Total	104	100
Health Condition		
Mental health	11	10.6
Physical health	13	12.5
Both	12	11.5
None	67	64.4
Prefer not to say	1	1.0
Total	104	100
Level of deprivation (SIMD)		
Q1 (most deprived)	10	9.6
Q2	12	11.5
Q3	16	15.4
Q4	19	18.3
Q5 (least deprived)	25	24.0
Unknown	22	21.2
Total	104	100
Currently pregnant		
No	100	96.2
Yes	4	3.8
Total	104	100
Number of previous pregnancies		
0	42	40.4
1	13	12.5
2	24	23.1
3	12	11.5
4	8	7.7
5 or more	5	4.9
Total	104	100

Around one third of participants (32.7%, *n* = 34) reported being involved in research as part of their occupation. Over one‐third of respondents worked as or had qualifications as a healthcare professional (37.5%, *n* = 39). Among respondents who reported working as a healthcare professional (*n* = 39), the most common profession was nursing (34.2%), followed by medical doctors (18.4%), with smaller proportions working in midwifery, allied health professions, and healthcare support roles. Forty per cent of participants (*n* = 42) reported that their current job role was associated with preconception health. Hence, 60% of respondents represented members of the general public.

Participants who reported that their role was associated with preconception health described involvement across several areas, including clinical care, public health practice, research, family support services, and health promotion activities. Clinical roles frequently involved providing contraception counselling, sexual health services, and maternity care, while others described population health initiatives, research on reproductive health, and community‐based support for families and parents.

#### 3.2.1 Preconception Health Familiarity and Importance

Participants reported moderate to high familiarity with the concept of preconception health (mean = 3.5, median = 4). Over half of respondents (55.1%) reported being very or extremely familiar with the concept.

Participants generally perceived preconception health as highly important, particularly for individuals planning a pregnancy (mean = 4.7, median = 5), women (mean = 4.6) and people of reproductive age (mean = 4.4). Importance ratings for men (mean = 4.1, ~78%–82% rating this as important) were comparable to those for individuals who were sexually active and not using contraception (mean = 4.1, ~80%–85%)—both somewhat lower than ratings for women, but still reflecting a majority view that preconception health matters for these groups. In contrast, lower importance ratings were reported for those using contraception (mean = 3.6, ~55%–60%) and, most notably, for individuals not planning a pregnancy (mean = 3.3, ~45%–50%), suggesting that perceived importance of preconception health remains closely tied to pregnancy intention and perceived risk of pregnancy.

As shown in Figure 3, the most commonly reported time for optimising health before pregnancy was 1 year before pregnancy, followed by 3 years, 3 months, 6 months and 5 years. A small number of respondents stated that they did not know when health optimisation should occur and 1% reported not before pregnancy.

**Figure 3:**
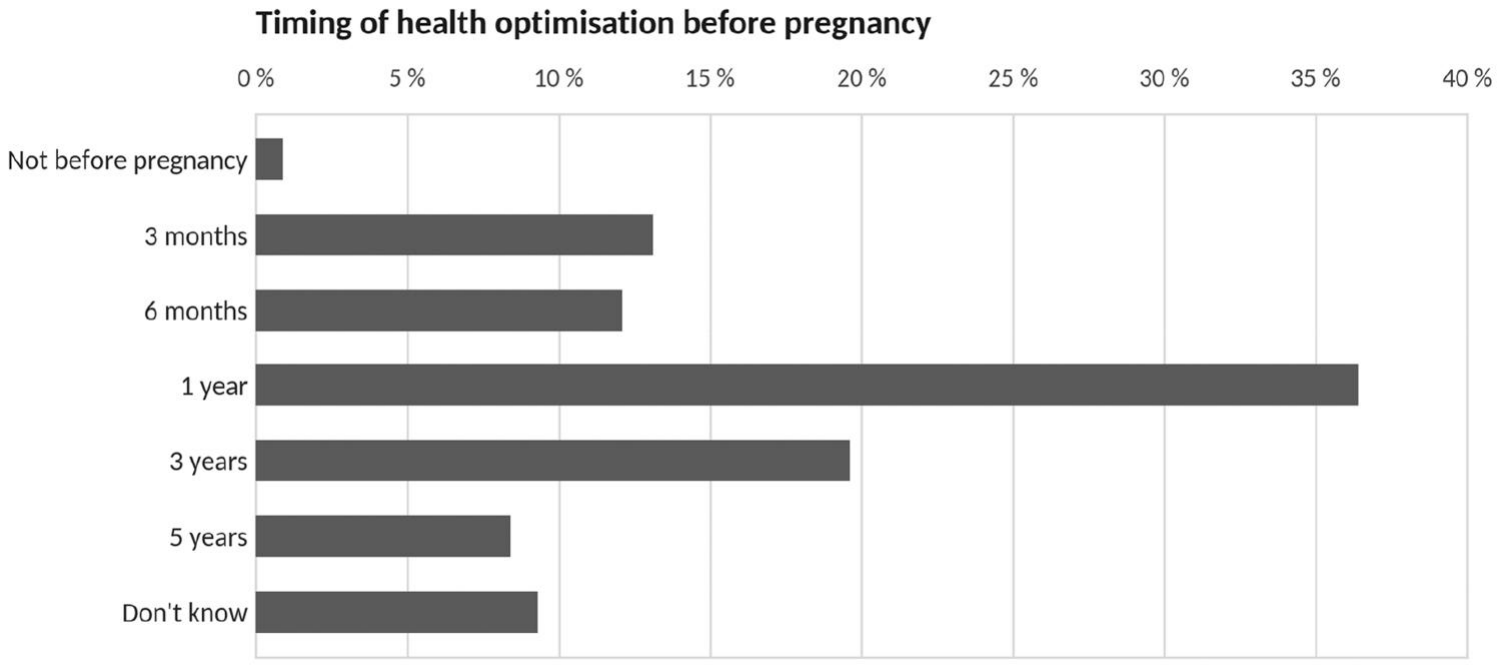
Graph showing participant views on ideal timing of health optimisation prior to pregnancy.

#### 3.2.2 Research Question Importance

Across items, most research questions were rated as important by a large majority of respondents. The highest ranked research priorities (based on the proportion rating the item as ‘important’) related to reducing neurodevelopmental risks and improving awareness of the impact of preconception behaviours, with 91% of respondents rating this question as important. An equal proportion prioritised improving preconception care for individuals with pre‐existing conditions (91%). Research addressing health inequalities and equitable access to preconception healthcare was also highly prioritised (88%–89%). Additionally, improving pregnancy planning and preparation behaviours was identified as a key research priority, with 87% of respondents rating these topics as important. The top six research questions from the survey are presented in Table 3.

**Table 3:** Top six research questions from the survey.

1	How can we reduce risks of babies having neurodevelopmental issues as well as increasing awareness of how behaviours before pregnancy impact risks of babies having neurodevelopmental issues?
2	How can preconception care be standardised or enhanced for those with pre‐existing conditions?
3	How do we promote and improve preconception health and wellbeing to everyone, regardless of the areas they live or their socio‐economic status?
4	How do we ensure that people have equal access to preconception health care, regardless of the areas they live or their socio‐economic status?
5	How can we promote and improve pregnancy planning?
6	How can we improve knowledge, intentions and behaviour of pregnancy planning and preparing for pregnancy?

When asked to select up to three priority areas for future research, the most frequently selected topic was a life course approach to women's and reproductive health (53.6%), followed by reducing neurodevelopmental risks and improving awareness of behavioural impacts before pregnancy (48.5%), and addressing health inequalities (47.4%). Research relating to men, partners and shared responsibility for preconception health was also commonly prioritised (42.3%). In contrast, fewer respondents selected topics relating to policy integration (11.3%), data collection (7.2%), or information provision (4.1%) as key research priorities. Figure 4 depicts priority themes.

**Figure 4:**
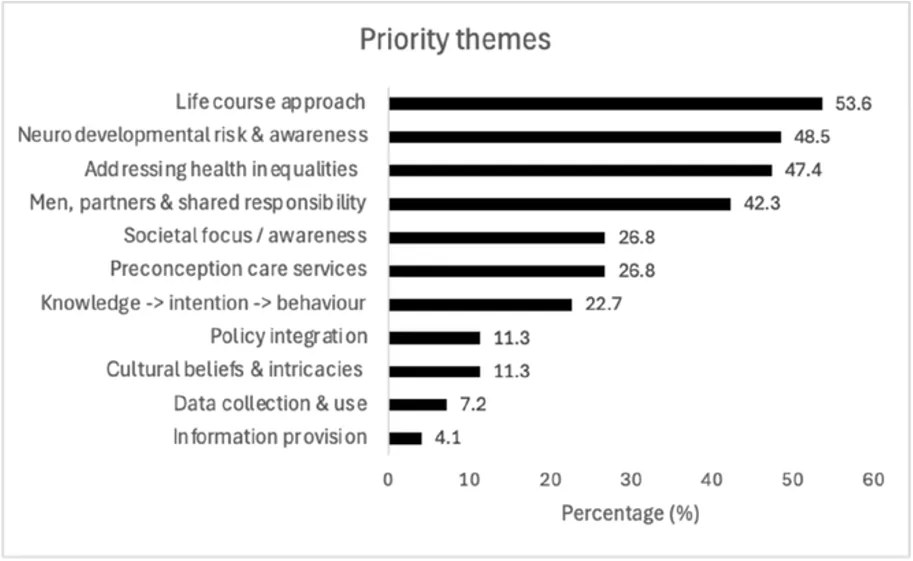
Priority themes from the survey.

Open‐text responses to the survey were also reviewed but did not identify themes beyond those already generated through the literature review and stakeholder consultation, and are therefore not reported in further detail. Hence, the top four priority themes (life course approach, neurodevelopmental risk and awareness, addressing health inequalities and men, partners and shared responsibility) were taken forward as a basis for the workshop.

### 3.3 Step Three: Workshop

Thirty‐eight people attended the in person workshop. Nine attended as members of the public with an interest in preconception health, 17 as part of their job role and the rest as ‘other’ which included students.

The workshop consisted of three rounds of activities where all participants had the opportunity to contribute to as many of the predefined themes as they wished and were asked to work in groups to develop and prioritise research questions within each theme. Group notes were taken during these discussions on flip chart paper by participant facilitators. These notes were then used by the research team (S.C., E.B., L.R.) to extract the prioritised research questions for each theme (Table 4) as well as some cross domain principles.

**Table 4:** Prioritised research questions from workshop.

1. Life course approach to preconception health How can early educational and behavioural intervention be consistently pitched across the lifespan? How can preconception health care be integrated into existing services and the wider public health lens? How can preconception care be standardised or enhanced for those with pre‐existing conditions? What are the most effective and accessible core preconception health messages for women and men in Scotland? How can we tailor interventions based on gender, life stage, ethnicity and community? What needs to be embedded in health care professional training to ensure and facilitate consistent core messages?
2. Neurodevelopmental risk and awareness What is the general awareness of neurodevelopmental risks and associated behaviours including folic acid supplementation, iodine supplementation and alcohol? What are the barriers to starting/stopping these behaviours? How can there be equitable access to resource/vitamins to reduce neurodevelopment risk? What are the best education and communication methods to promote awareness? What are the most effective healthcare pathways to address risk and awareness? How can inter‐conception be capitalised to reduce risk and increase awareness? What is the impact of policy change and intervention to health outcomes and behaviour?
3. Addressing health inequalities In what ways can inclusive access to preconception services, resources and awareness be optimised? What are the indicators which represent preconception health inequalities? How can we explore, understand and monitor these? How can interventions be culturally appropriate without stereotyping? In what ways can local environments be optimised to become ‘healthy communities'? Who is currently underrepresented in preconception health research and interventions and how can this be addressed?
4. Men, partners and shared responsibility What are the main barriers to male inclusion in preconception care? What effective communication strategies target and include men/partners? Considering language, framing, inclusion, context… What are men's behavioural risk awareness? What are the social influences on preconception health in men?

A number of cross domain principles were identified as important to consider in all future preconception health research in Scotland. These were:

- Consistent messaging and communication which is evidence based and tailored
- Inclusivity and accessibility
- Health care professional training and support
- Life course importance
- Scope of digital health and resource to promote and support preconception health and care
- Stakeholder involvement importance in research
- Measurable outcomes and importance of high quality data collection

The produced artistic piece can be seen in Figure 5 and will be used to promote preconception health at a range of stakeholder events.

**Figure 5:**
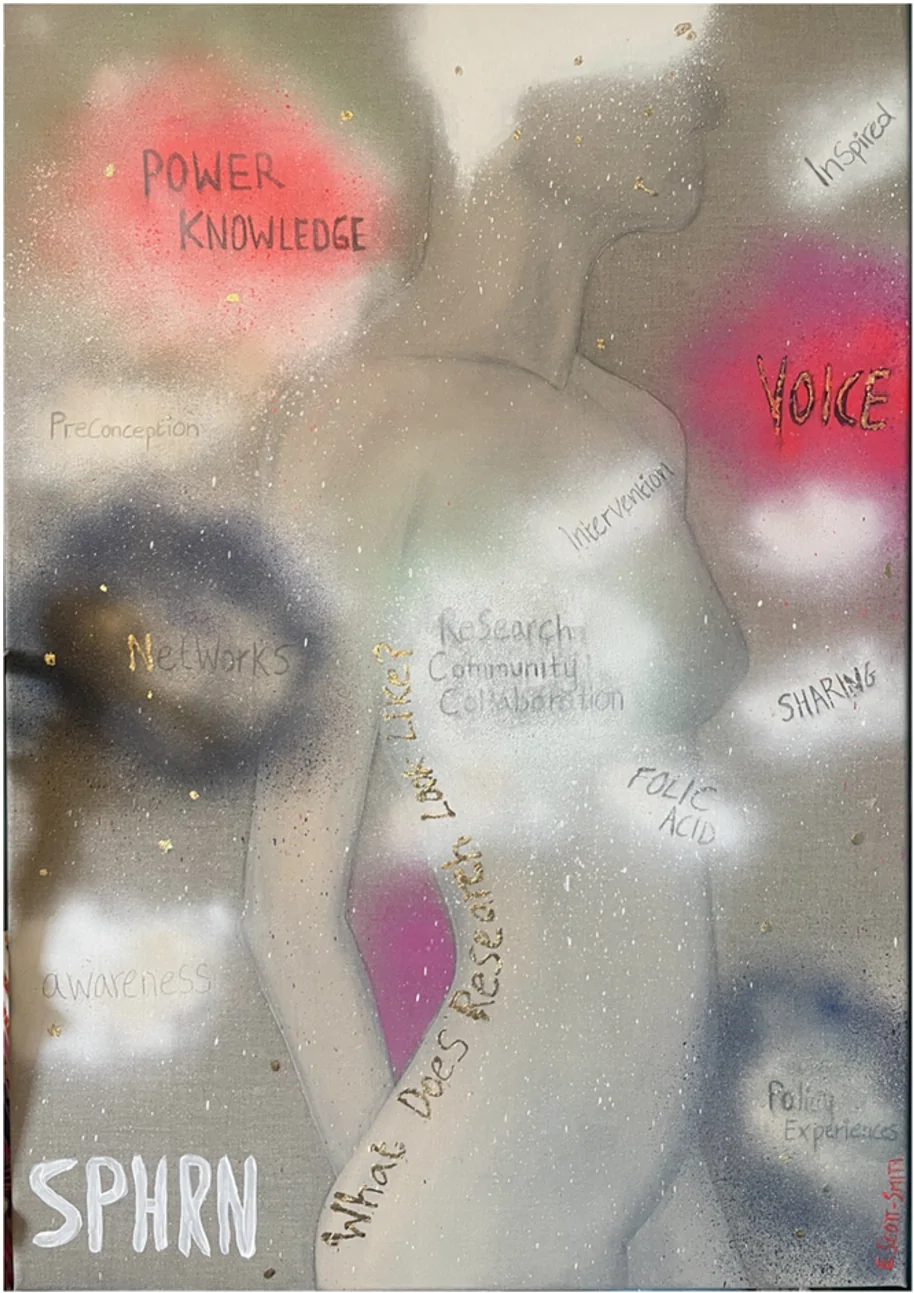
Visual depiction of workshop discussions entitled ‘Preconception health and science in research’ created live by independent artist, Emma Scott‐Smith, (www.emmascott-smith.com).

## 4 Discussion and Call to Action

This research prioritisation project resulted in prioritised themes and research questions for preconception health research in Scotland co‐designed by stakeholders. There are four research themes, 21 research questions and seven core, over‐arching principles which should be integrated into all preconception health research in Scotland. Whilst this does not represent the vast array of required research, it prioritises the key areas which are timely and essential to support the growing political and health interest in preconception health in Scotland. Advancements in these research areas can help governments, policymakers, national health services, education sectors, economies, individuals, and families optimise health and outcomes for future generations.

Taking a life course approach to health is recognised widely, initially by Barker et al. [5] and subsequently championed by public health agencies across the United Kingdom, including Scotland. A life course approach is seen as a preventative strategy to reduce ill health and disease in the long term. To ensure that the current interest from government and public health is optimised, high quality, context‐specific research is needed to facilitate evidence based practice and policy. Our prioritised research themes and questions will facilitate this.

Whilst there have been some developments in systems level actions to mitigate neurodevelopmental risks, in the form of folate fortification of flour [42, 43], there have been concerns that these are not enough or adequate to reduce risk of preventative neurodevelopmental issues [44]. Hence, there is a pertinent need for research in this area to quantify and implement the best actions for appropriate reduction in risk specifically related to preconception and long‐term child health.

As also found by UK Preconception Partnership [45], there is clear evidence that health inequalities and disparities need to be addressed in all areas of preconception health research. Scotland has some of the worst deprivation and health inequalities in the United Kingdom. For example, the gap in healthy life expectancy is 24 years between the most and least deprived women in Scotland and life expectancy in the poorest areas has decreased since 2010 [46]. Women living in deprived areas are more likely to be exposed to health harms (e.g., alcohol consumption, drug use, poor housing) and less likely to access preventative healthcare, compared to those in more affluent areas [47]. These deep‐seated inequalities also require a wider, systems‐based approach with moral, political and economic determinants investigated and actioned to resolve and improve the health of current and future generations. Future priority‐setting work should also consider how underserved populations and marginalised communities can be more directly engaged, rather than relying on survey and workshop participation alone as the route to representation. While every effort was made to ensure a broad range of voices were heard in this exercise, it is possible that the prioritisation process itself underrepresented some groups, reflecting who was reached by, and who chose to take part in, the survey and workshop. Community champion models, in which trusted members of a community lead engagement rather than researchers or academics, may offer a more inclusive alternative to a primarily research‐driven approach in future work of this kind. These considerations align closely with the aims of the Maternity Disparities Taskforce [48], established to address unacceptable disparities in maternity outcomes, which could offer a useful model, or potential partner, for future preconception health prioritisation work with underserved groups.

The specific themes and priorities identified in this project should be interpreted in light of who took part. Survey respondents were highly educated, with 42% living in the least deprived areas of Scotland; demographic data on workshop attendees was not systematically collected. This pattern of engagement may partly explain both the strength of the health inequalities theme, reflecting an awareness among participants of who was, and was not, represented in the process, and the relative emphasis on knowledge‐ and awareness‐based research questions over more immediate practical barriers that may be more salient to less advantaged groups. Future prioritisation work should collect and report participant demographics more comprehensively at every stage, including workshops, to better understand how engagement shapes outputs.

Comparison with existing UK priority‐setting exercises in preconception health suggests some convergence in how needs are structured, alongside important differences in scope and emphasis. A recent UK‐wide consensus study identified 16 priority topics for preconception care in general practice, consolidated into four overarching areas: patient knowledge, ideas/concerns/expectations, health conditions, and health behaviours [8]. As in the current project's findings, this points to the value of organising priorities into a small number of overarching themes; however, the focus of Schoenaker et al. [49] was clinical, aimed at supporting opportunistic preconception care within routine general practice consultations, whereas our themes and questions span policy, systems and research need more broadly across the life course. Notably, Schoenaker et al. [49] also found that people of reproductive age and practitioners prioritised different topics (infertility and mental health vs*.* weight and folic acid supplementation, respectively), echoing our own reflection on how participant demographics may shape which priorities emerge. Similarly, [50] international prioritisation project on preconception health and maternal obesity identified overarching principles including social determinants, family health and cultural context, aligning closely with our own overarching research principles. Together, these comparisons suggest reasonable consistency in how preconception health priorities are structured across settings and methods, even where scope and target audience differ, and indicate value in future work systematically comparing prioritisation outputs across preconception research, policy and care delivery internationally.

A notable finding from the narrative review was that, while UK evidence informed preconception health policy and guidance broadly, none of the reviewed literature focused specifically on Scotland, despite Scotland experiencing some of the most pronounced health inequalities in the United Kingdom. This is consistent with Cassinelli et al.'s [34] scoping review of preconception health policy across the United Kingdom and Ireland, which found limited country‐specific analysis for Scotland and identified a limited focus on health inequalities as a key gap in existing resources—a gap this project addresses directly. Our research priorities are grounded in Scottish policy and shaped by Scottish stakeholders and services, rather than assuming priorities identified elsewhere in the United Kingdom or internationally translate directly to the Scottish context. This is particularly relevant given Scotland's distinct devolved health policy landscape and the existence of dedicated infrastructure, such as the Scottish Preconception Health Research Network, through which these priorities can now be taken forward.

Finally, the priority theme of men, partners and shared responsibility is highly topical across the world. The involvement of men and partners in both the biological and behavioural components of preconception health have been recently emphasised by Huang et al. [7] who concluded that greater attention is needed on young men to facilitate more equitable and holistic preconception health interventions and policy. Furthermore, recent review found that men are still underrepresented in preconception care, and that many have low fertility awareness and inconsistent healthy preconception behaviours [51]. Therefore, prioritising research with men and partners to enhance shared responsibility in Scotland is well justified.

We have also highlighted some clear overarching research principles which relate to all research themes and emphasise the multidisciplinary nature of this topic area, as well as the population‐level importance. These include consistency, inclusivity, stakeholder involvement and high‐quality data. We call on researchers across Scotland (and beyond) to work collaboratively to advance contextually relevant preconception health research. For researchers, these themes and questions offer a clear, evidence‐informed agenda that can guide study design and reduce duplication of effort, particularly once refined further using frameworks such as PICO. For policymakers, having Scotland‐specific priorities, rather than relying on evidence generalised from elsewhere in the UK or internationally, provides a stronger basis for embedding preconception health explicitly within strategies such as the Women's Health Plan phase two. For funders, this prioritisation exercise offers a mandate for targeted funding calls in areas of genuine need, helping to direct limited research funding towards questions that are not already well evidenced elsewhere. Looking ahead, these priorities could underpin a coordinated national research programme for preconception health in Scotland, for example, through targeted funding calls, PhD and fellowship opportunities, and continued collaboration through the Scottish Preconception Health Research Network, ensuring that research effort builds cumulatively towards the outcomes outlined above rather than progressing in a fragmented way. Pursuing and exploring the research themes and questions prioritised in this project is essential in order to:

− improve physical and mental health before, during and after pregnancy,
− improve long‐term child health outcomes,
− support the emerging health care pathways and health care professionals who deliver preconception health care,
− reduce health inequalities in reproductive age adults and children
− help funders and policymakers make informed decisions about the shorter‐ and longer‐term preconception health research priorities to align with the Scottish population need.

The prioritised research themes, questions and overarching principles were generated through a robust process based on gold standard research principles [52], [21] as well as utilising and applying published literature, Scottish policy and government guidance documents, anonymised data collection and diverse stakeholder engagement. Participants and stakeholders were diverse in discipline and research experience where over two‐thirds of participants did not work in preconception health‐ related fields, hence representing the general public. Many of the prioritised research themes and questions can be promptly addressed, with minimal investment from research fundings bodies, health boards and/or government. For example, existing and ongoing developments related to The Women's Health Plan phase two [17] can consider how preconception health care is delivered and integrated into existing services in Scotland. Whilst funding and resources are needed to answer the priority research questions, there is strong alignment with ongoing activities hence, minimal investment will result in research answers and long‐term outcomes.

Some priorities lend themselves to more immediate action than others. Questions relating to service delivery and integration, such as how preconception care is delivered within existing pathways, can be progressed through alignment with ongoing policy developments such as the Women's Health Plan phase two, whereas questions requiring new longitudinal data collection or intervention testing will necessarily take longer and require dedicated funding. Addressing the prioritised themes will also depend on interdisciplinary collaboration across primary care, public health, reproductive health services and the third sector, and on stronger partnerships between researchers and policymakers to ensure findings translate into practice and guidance. Where new infrastructure is needed, this may include shared data systems to support longitudinal follow‐up, dedicated preconception care roles or pathways within primary care, and sustained funding mechanisms that allow research and service development to progress together rather than sequentially.

Limitations of this project include self‐selection of participants and stakeholders to complete the survey or attend the workshop. Despite every effort to advertise the survey via diverse community links and facilitate attendance at the workshop with funding for time and travel, the sample was still highly educated. This may mean the results are skewed and not fully representative of the whole Scottish population. Additionally, no participants identified as Black, African or Caribbean, which should be considered when interpreting the representativeness of the demographic profile reported. However, this is a common limitation with preconception health research, as a recent citizens panel gathering views on preconception health and care by Healthcare Improvement Scotland (2005), found similarly skewed participant demographics. Additionally, step one was based on a small non‐systematic review of reviews and some more recent, emerging literature not yet included in reviews may not have been included.

All themes and questions generated remain unanswered in a Scottish context by existing research, reflecting research need for preconception health research in Scotland. Further work to refine each research question (e.g., using the Population, Intervention, Comparator, Outcome framework) [53] would enable groups of multidisciplinary researchers to answer them effectively.

## Author Contributions

**Sinead Currie:** conceptualisation,methodology,investigation, supervision, funding acquisition, project administration, writing – original draft, writing – review and editing. **Emma J. Brough:** conceptualisation, formal analysis, funding acquisition, investigation, methodology, writing – original draft, writing – review and editing. **Laura Ryan:** investigation, methodology, project administration, writing – review and editing. **Kathleen Renwick:** investigation, methodology, project administration, writing – review and editing. **Beth Rose:** investigation, methodology, writing – review and editing. **Annie Ganguly:** investigation, methodology, writing – review and editing.

## Ethics Statement

Ethical approval was granted from the University of Stirling General Ethics Panel (GUEP, 22682).

## Conflicts of Interest

The authors have no conflicts of interest.

## Supporting information


Supporting File 1

Supporting File 2

Supporting File 3

## Data Availability

The data that support the findings of this study are available from the corresponding author upon reasonable request.
