# Supplementary material for: Research Priorities for Preconception Health in Scotland: A Multidisciplinary Call to Action
Source: Health Expect. 2026 Oct 29;29(6):e70915. doi: 10.1111/hex.70915 (PMC13717299; doi:10.1111/hex.70915)
Supplement: Supplementary file 1 [file HEX-29-e70915-s001.docx]

| Section and topic | Item | Reported on page No |
| --- | --- | --- |
| 1: Aim | Report the aim of PPI in the study | 4 |
| 2: Methods | Provide a clear description of the methods used for PPI in the study | 6-8 |
| 3: Study results | Outcomes—Report the results of PPI in the study, including both positive and negative outcomes | 10-17 |
| 4: Discussion and conclusions | Outcomes—Comment on the extent to which PPI influenced the study overall. Describe positive and negative effects | 18-22 |
| 5: Reflections/critical perspective | Comment critically on the study, reflecting on the things that went well and those that did not, so others can learn from this experience | 21 |

GRIPP2 short form

PPI=patient and public involvement
