# Supplementary material for: Research Priorities for Preconception Health in Scotland: A Multidisciplinary Call to Action
Source: Health Expect. 2026 Oct 29;29(6):e70915. doi: 10.1111/hex.70915 (PMC13717299; doi:10.1111/hex.70915)
Supplement: Supplementary file 2 [file HEX-29-e70915-s002.docx]

**Appendix 2**

**Workshop Agenda**

Morning

Welcome and setting the scene for preconception health

Table introductions

Short research presentations: Current preconception health research in Scotland

Panel Q&A round up

Afternoon

Introduction to afternoon session

Research development workshop

Topics will include:

Life course approach to preconception health

Neurodevelopmental risk and awareness

Addressing health inequalities

Men partners and shared responsibility

Other

Consolidation of workshop, summary, next steps and close
