# Supplementary material for: Research Priorities for Preconception Health in Scotland: A Multidisciplinary Call to Action
Source: Health Expect. 2026 Oct 29;29(6):e70915. doi: 10.1111/hex.70915 (PMC13717299; doi:10.1111/hex.70915)
Supplement: Supplementary file 3 [file HEX-29-e70915-s003.docx]

**Appendix One.**

**Step 2: Survey**

**Section 1**

Please tell us a little about yourself. This helps us make sure we are reaching a wide range of people and to better understand the research needs from different groups of people.

1. What was your age on your last birthday?
2. What is your home postcode?
3. What was your sex at birth?

male, female, prefer not to say

4. What gender do you identify with now?

Male/ female/ non-binary/ prefer not to say/ other

5. What is your sexual orientation?

heterosexual/ straight, gay/ lesbian, bisexual, prefer not to say, prefer to describe___

6. What is your ethnic group (as per Scottish census)? Please choose one section from A to F, then tick one box which best describes your ethnic group or background

A. White

1. Scottish

2. Other British

3. Irish,

4. Polish

5. Gypsy / Traveller

6. Roma

7. Showman / Showwoman

8. Other white ethnic group, please write in:

B. Mixed or multiple ethnic groups

Any mixed or multiple ethnic groups, please write in:

C. Asian, Scottish Asian or British Asian

1. Pakistani, Scottish Pakistani or British Pakistani

2. Indian, Scottish Indian or British Indian

3. Bangladeshi, Scottish Bangladeshi or British Bangladeshi

4. Chinese, Scottish Chinese or British Chinese

5. Other, please write in:

D. African, Scottish African or British African

Please write in (for example, Nigerian, Somali):

E. Caribbean or Black

Please write in (for example, Scottish Caribbean, Black Scottish):

F. Other ethnic group

1. Arab, Scottish Arab or British Arab

2. Other, please write in (for example, Sikh, Jewish):

G. Other not listed _____

H. prefer not to say

7. What is your highest level of qualification?

Degree level or higher qualifications; further education qualifications such as HNC, HND SVQ level 4; apprenticeship qualifications; upper school qualifications such as Highers, A levels; lower school qualifications such as standard grades, GCSE; no qualifications

8. Do you have any physical or mental health conditions or illnesses lasting or expected to last 12 months or more?

Yes/ No/ Prefer not to say

9. What is your current employment status?

Working as an employee; Self-employed or freelance; Temporarily away from work ill, on holiday or
temporarily laid off; On maternity or paternity leave; Doing any other kind of paid work; None of the above

10. Are you currently pregnant/ is your partner currently pregnant?

Yes/no

11. How many pregnancies have you experienced/ experienced with a partner? open
12. (filter only if yes to Q11) How many biological children do you have? Open
13. (filter only if yes to Q11) How old were you when you first experienced a pregnancy (either yourself or with a partner)?
14. If currently employed, what is your job role/ occupation?

Open ended:

15. If currently employed, What type of organisation do you work for?
    - Education including schools
    - Higher education including university
    - NHS
    - Third sector/ charity
    - Private business
    - Other (please specify)
16. Are you a researcher or are you involved in research as part of your occupation? Yes/ no
17. Do you work or have qualifications as a health care professional? Yes/ no. If yes, what type of HCP?
18. Do you see your job role/occupation associated with preconception health in some way? If yes, please explain how
19. Do you have any non-paid roles? If yes, please specify
20. (only if yes to Q19) do you see your unpaid role(s) being associated with preconception health in some way? If yes, please explain how

As a reminder, preconception health means the health and wellbeing of people (any gender) during their childbearing years (approximately age 16 to 50).

21. How familiar are you with the idea of preconception health/ health and wellbeing before pregnancy? (Likert scale)
22. What does preconception health and wellbeing before pregnancy mean to you? (open ended)
23. How important do you think preconception health is for (Likert scale 5 point not important at all, extremely important):
    - Men
    - Women
    - People of reproductive age (approx. 16-49)
    - Those planning a pregnancy
    - Those not planning a pregnancy
    - Those who are sexually active and using contraception
    - Those who are sexually active and not using contraception
24. When do you think is the best time for people to optimise health before pregnancy?
    - at least 5 years before pregnancy,
    - at least 3 years before pregnancy,
    - at least 1 year before pregnancy,
    - at least 6 months before pregnancy,
    - at least 3 months before pregnancy,
    - not before pregnancy,
    - Don't know
25. Please tell us why you think preconception health and wellbeing before pregnancy is important or not.

Section 2

The research team have looked at a wide range of research papers, Scottish policies and other documents about preconception health. Using this information, we have come up with a list of research questions relevant to preconception health, specifically in Scotland. We conduct research (i.e. collect information about people and services through surveys, interviews, data sets etc) to answer research questions. These answers then give information and evidence to (hopefully) improve health outcomes, experiences, services and other things. We will present these to you now and will ask you:

1) to tell us how important you think each question is,

2) if you have any ideas for research questions

3) to pick your top 3 areas which, you think, are most important to be researched.

Before we present the list of research questions, please tell us, in your opinion, what are the three most important research areas/topics/questions related to preconception health and wellbeing (open ended)

Please tell us how important you think each of these research questions are for preconception health and wellbeing in Scotland. There are no right or wrong answers, we are interested in your own opinions and thoughts (scale: important, neutral, not important. questions will be presented in a random order)

1. What are men’s preconception health knowledge levels, behaviours, intentions, and needs?
2. How can the concept of shared responsibility for preconception/ reproductive health be promoted and improved?
3. How does cultural diversity^[1]^ in Scotland influence preconception health?
4. How can cultural diversity be supported in preconception health promotion and care?
5. What are people of Scotland’s preferences for receiving preconception care? Including what information do they want and what support is needed?
6. How can preconception health care be delivered in Scotland? including where, by who, to who, and how to promote accessibility?
7. What are health care professionals responsibilities and training needs for providing preconception health care?
8. Should preconception care be integrated into reproductive and sexual health services, if so how?
9. Is a preconception health care pathway needed in the NHS and what should this look like? What does it include e.g. mental health? Physical health?
10. How can preconception care be optimised/ standardised for those with pre-existing conditions such as mental health, diabetes, CVD or other chronic conditions?
11. Can provision of early education for people of reproductive age improve informed decision making around pregnancy planning/ timing?
12. How do we ensure that people have equal access to preconception health care, regardless of the areas they live or their socio-economic status? This could include access to reproductive health services, primary care...
13. How do we promote and improve preconception health and wellbeing to people, regardless of the areas they live or their socio-economic status? This could include education, improving health behaviours such as diet and smoking...
14. What type of education is needed to educate people about preconception health and associated reproductive issues including conception, fertility, ovulation? how can this be provided to all ages regardless of pregnancy intent (i.e. anyone who is sexually active)
15. How do can public awareness of preconception health be improved?
16. What are societal norms and education around preconception health and how can these be improved?
17. How can preconception health be promoted and integrated into settings outside of healthcare?
18. How can awareness of epigenetics^[2]^ and personal control be promoted?
19. How can we promote and improve pregnancy planning?
20. How can online preconception health information be optimised? content, access, location, signposting...
21. How can preconception health be integrated into a life course approach to women's health/ reproductive health? from individual to societal level?
22. How can the Flour fortification legislation^[3]^ be promoted to allow for appropriate interpretation and avoidance of unintended consequences?
23. How to ensure preconception health is integrated into any child health policies?
24. What preconception health messages should all NHS boards be providing and how are these delivered/implemented consistently? (including clinical guidelines and responsibilities)
25. Can preconception health education be provided at all ages regardless of pregnancy intent (i.e. anyone who is sexually active)?
26. How can we improve knowledge, intentions and behaviour of pregnancy planning/ optimisation?
27. How can we improve knowledge, intentions and behaviour of Folic acid supplementation?
28. How can we improve knowledge, intentions and behaviour of Iodine supplementation?
29. How can we improve knowledge, intentions and behaviour of Healthy weight/BMI including physical activity and diet?
30. How can we improve knowledge, intentions and behaviour of Smoking cessation?
31. How can we improve knowledge, intentions and behaviour of Alcohol cessation?
32. How can we improve knowledge, intentions and behaviour of Drug use/ addictions cessation?
33. How can we reduce risks of neurodevelopmental issues and increase awareness of preconception behavioural impact on neurodevelopmental issues? This includes issues such as FASD...
34. What are the preconception health indicators (risk factors) in scotland?
35. How can we optimise data collection of preconception health data?
36. What are the long term patterns and outcomes of preconception health behaviours across Scotland?

Are there any other research questions you think should be asked which are not covered here?	(open ended)

Using the list of research questions above, we have grouped these into 11 topic areas. These are listed below. Please choose up to three topics which you think are most important to be researched.

- Men, partners and shared responsibility for preconception health
- Exploring, addressing and optimising cultural beliefs and intricacies
- Preconception care services
- Addressing and reducing health inequalities
- Societal focus/impact/awareness on PC health
- Information Provision (how/what information about preconception health is provided)
- life course approach to women's health/ reproductive health
- Policy integration and generation
- Facilitating the transition from knowledge to intention to behaviour
- How can we reduce risks of neurodevelopmental issues and increase awareness of preconception behavioural impact on neurodevelopmental issues?
- How to collect and use data most effectively

At the start of this survey we asked you to tell us, in your opinion what are the three most important research areas/topics/questions related to preconception health and wellbeing. Please tell us again what you think the three most important research areas/topics/questions are related to preconception health and wellbeing. This might be the same or different to what you listed at the start of the survey (open ended)

Other research needs:

- Do you have any ideas or suggestions on how to tell others about the findings from this project? E.g. who might be interested to know about the research needs and priorities (and subsequent research findings) and how do we tell them?
- Would you like to be invited to help shape or take part in any relevant research on the topic of preconception health in the future? Yes/ no. If yes, please provide your name and email address and/or telephone number on this form [link to MS forms].

[end]

1. defined as: the variety of cultural expressions, practices, and identities that exist within a society or community. It encompasses the different backgrounds, languages, traditions, and beliefs that shape individuals and groups.
2. definition of epigenetics: how our environment influences our genes by changing the chemicals attached to them, for example how what we eat, our physical activity level, access to resources and more affect those chemicals, in turn shaping our health.
3. [Bread and flour to be fortified with folic acid to prevent birth defects | Food Standards Scotland](https://www.foodstandards.gov.scot/news-and-alerts/bread-and-flour-to-be-fortified-with-folic-acid-to-prevent-birth-defects)
